# Integrative Analysis of 5-Hydroxymethylcytosine and Transcriptional Profiling Identified 5hmC-Modified lncRNA Panel as Non-Invasive Biomarkers for Diagnosis and Prognosis of Pancreatic Cancer

**DOI:** 10.3389/fcell.2022.845641

**Published:** 2022-03-25

**Authors:** Shuangquan Li, Yiran Wang, Caiyun Wen, Mingxi Zhu, Meihao Wang, Guoquan Cao

**Affiliations:** ^1^ The First School of Medicine, School of Information and Engineering, The First Affiliated Hospital of Wenzhou Medical University, Wenzhou, China; ^2^ Department of Anatomy, School of Basic Medicine and Life Science, Hainan Medical University, Haikou, China

**Keywords:** 5-hydroxymethylcytosine, pancreatic cancer, machining learning, long non-coding RNA, non-invasive biomarker

## Abstract

Pancreatic adenocarcinoma (PAAD) is the fourth leading cause of cancer-related deaths worldwide. 5-Hydroxymethylcytosine (5hmC)-mediated epigenetic regulation has been reported to be involved in cancer pathobiology and has emerged to be promising biomarkers for cancer diagnosis and prognosis. However, 5hmC alterations at long non-coding RNA (lncRNA) genes and their clinical significance remained unknown. In this study, we performed the genome-wide investigation of lncRNA-associated plasma cfDNA 5hmC changes in PAAD by plotting 5hmC reads against lncRNA genes, and identified six PAAD-specific lncRNAs with abnormal 5hmC modifications compared with healthy individuals. Then we applied machine-learning and Cox regression approaches to develop predictive diagnostic (5hLRS) and prognostic (5hLPS) models using the 5hmC-modified lncRNAs. The 5hLRS demonstrated excellent performance in discriminating PAAD from healthy controls with an area under the curve (AUC) of 0.833 in the training cohort and 0.719 in the independent testing cohort. The 5hLPS also effectively divides PAAD patients into high-risk and low-risk groups with significantly different clinical outcomes in the training cohort (log-rank test *p* = 0.04) and independent testing cohort (log-rank test *p* = 0.0035). Functional analysis based on competitive endogenous RNA (ceRNA) and enrichment analysis suggested that these differentially regulated 5hmC modified lncRNAs were associated with angiogenesis, circulatory system process, leukocyte differentiation and metal ion homeostasis that are known important events in the development and progression of PAAD. In conclusion, our study indicated the potential clinical utility of 5hmC profiles at lncRNA loci as valuable biomarkers for non-invasive diagnosis and prognostication of cancers.

## Introduction

Pancreatic adenocarcinoma (PAAD) is the fourth leading cause of cancer-related deaths worldwide (Siegel et al., 2020). The lack of early-stage diagnostics has hindered the development of therapeutics that can slow down or reverse PAAD (Moutinho-Ribeiro et al., 2017; Sohal et al., 2017). Carbohydrate antigen 19–9 (CA19-9) is the biomarker currently used for PAAD diagnosis (Poruk et al., 2013). However, CA19-9 has a pooled sensitivity of 75.4% (95% CI: 73.4–77.4%) and a specificity of 77.6% (95% CI: 75.4–79.7%) for differentiation between malignant and non-malignant forms of cancer (Zhang et al., 2015). Moreover, the specificity of distinction between PAAD and CP often does not exceed 60% (Schultz et al., 2014), which has prompted a search for alternative biomarkers.

Circulating cell-free DNA (cfDNA) originates from cell death in different tissues, which has attracted massive interest as a non-invasive biomarker for cancer detection (Wan et al., 2017). Tumor cells release small nucleic acid fragments into the blood *via* multiple mechanisms, allowing cancer-associated genetic alterations to be detected (Diaz and Bardelli, 2014; Wan et al., 2017). Non-invasive biomarkers offer substantial advantages over tissue biopsy as their easily accessible characteristics make them ideal candidates for cancer diagnosis and progression monitoring (Xu et al., 2017).

5-methylcytosine (5mC) modifications potentially characterized various health conditions (Lehmann-Werman et al., 2016; Guo et al., 2017). Nonetheless, there has been no investigation to identify and sequence alternative modifications in circulating cfDNA because the DNA samples are low-input. 5-Hydroxymethylcytosine (5hmC) is a novel identified epigenetic mark generated from 5mC by the ten-eleven translocation proteins (Tahiliani et al., 2009). Increasing evidence showed that low levels of 5hmC are observed in many tumors frequently compared to corresponding normal tissues (Jin et al., 2011). 5hmC is a stable intermediate of cytosine demethylation. Active gene modification was associated with the levels of 5hmC accumulation in promoters, gene bodies and gene regulatory elements (Han et al., 2016). 5hmC modifications play a curial role in cell development, differentiation, maturation and self-renewal (Wang et al., 2014). These characteristics suggested 5hmC signaling changes in cfDNA may have potential values in cancer diagnosis and progression monitoring using highly robust and sensitive 5hmC sequencing technologies (Song et al., 2017).

Long non-coding RNA (lncRNA) expression is the most pervasive transcriptional change in cancer, which is demonstrated by the recent genome-wide characterization of the human cancer transcriptome (Marchese et al., 2017; Zhou et al., 2019a; Bao et al., 2020; Zhou et al., 2021b). Multiple studies have shown that lncRNAs play a critical role in the mechanism of occurrence, evolution, invasion and metastasis of pancreatic cancer (Fu et al., 2017; Zhang et al., 2020; Tang et al., 2021). Several studies have indicated the impact of lncRNAs on the prognosis of pancreatic cancer (Huang et al., 2016; Zhou et al., 2016). There is increasing evidence that lncRNA expression could also be regulated by epigenetic DNA modifications, such as DNA methylation, histone modifications and 5hmC modification (Guttman et al., 2009; White et al., 2014; Yan et al., 2015). Epigenetic DNA modifications in lncRNA genes have been revealed to be valuable non-invasive biomarkers for cancer diagnosis, prognosis and surveillance (Hu et al., 2017; Zhou et al., 2021a).

In the present study, we performed machine learning-based integrative analysis of 5-hydroxymethylcytosine and transcriptional profiling to identify plasma-derived lncRNAs with PAAD-specific abnormal 5hmC modifications and conducted a two-phase discovery-validation experiment to explore the clinical potential of 5hmC-modified lncRNAs as non-invasive biomarkers for diagnosis and prognosis of pancreatic cancer.

## Materials and Methods

### Sample Datasets

**FIGURE 1:**
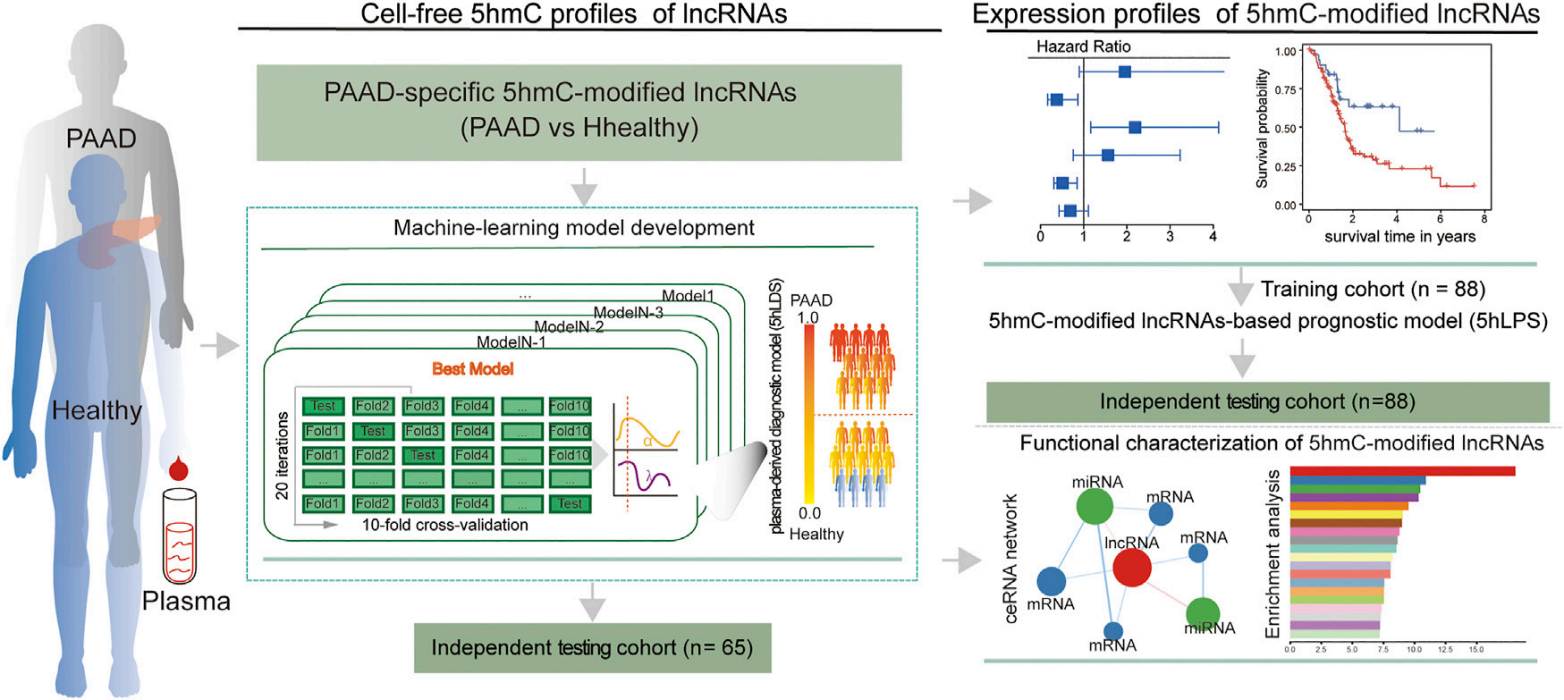
Workflow diagram of the study design. A two-phase discovery-validation study was conducted. The 5hmC-modified lncRNA-based predictive and prognostic models for PAAD were developed using the machine-learning methods in the training cohort and validated in the independent testing cohort. PAAD, Pancreatic adenocarcinoma; lncRNA, long non-coding RNAs; 5hmC, 5-Hydroxymethylcytosine.

A total of 130 publicly available genome-wide nano-hmC-Seal profiles from plasma cfDNA samples were used in this study, including 34 PAAD patients and 96 healthy individuals from Sequence Read Archive (SRA, SRP080977) of the National Center for Biotechnology Information (NCBI) (Li et al., 2017). Clinical and transcriptomic data (RNA-Seq, v.2019-07-20) of 182 PAAD cases, including 176 primary tumors and four solid tissue normal, generated by The Cancer Genome Atlas (TCGA) were downloaded from UCSC Xena (https://xena.ucsc.edu/). In addition, stem-loop expression (miRNA, v.2019.7.20) of 183 PAAD cases, including 1881 miRNAs, was also obtained from UCSC Xena. Detailed information on the study population is shown in Table 1. The workflow diagram of the study design is shown in Figure 1

**TABLE 1:** Baseline clinical and pathological characteristics of the study population.

Cohorts	Sample type	Number (%)	Platform	Data source
Li’s cohort	Pancreatic Cancer	130	nano-hmC-Seal-seq	SRP080977
		34		
	Healthy	96		
TCGA-PAAD		180	RNA-seq	TCGA
	Primary Tumor	176		
	Solid Tissue Normal	4		

### Data Preprocessing and Mapping of 5hmC-Modified lncRNAs

Read sequences were extracted in FASTQ files using the SRA toolkit (https://trace.ncbi.nlm.nih.gov/Traces/sra/sra.cgi?view=software, version 2.9.2), and then were aligned to the human genome GRCh37 using Bowtie2 (version 2.3.4.2) with default parameters (Langmead and Salzberg, 2012). To convert and sort the alignment SAM files into BAM files, SAMtools (version 1.9) was used to generate the files (Li et al., 2009). The picard-2.18.4 was used to retain unique non-duplicate matches to the genome (http://broadinstitute.github.io/picard/). The released version of the lncRNA reference gene annotation file (GRCh38 version 34) was downloaded from the GENCODE database (https://www.gencodegenes.org/). LiftOver was used to transfer the mapping information from the GRCh38 version of the lncRNA reference gene annotation file to the GRCh37 version. Genes encoding lncRNAs were extracted based on GRCh37 annotation. Read counts of 5hmC-modified lncRNAs were calculated using the fragment counts in each RefSeq lncRNA obtained by BEDtools (version 2.27.1) (Quinlan and Hall, 2010). The read counts were converted into Transcripts Per kilobase of 5hmC in lncRNA per million mapped reads. Finally, 5hmC profiles of 16,827 lncRNAs were obtained for further analysis.

### Machining Learning-Based Establishment of a Non-Invasive Diagnostic Model Based on 5hmc Modified lncRNAs

The 5hmC profiles of lncRNAs were compared between PAAD and healthy control samples. The lncRNAs with differential 5hmC modification were identified using the DESeq2 package (version 1.22.2) (Love et al., 2014) with a |log2foldchange|>0.58 and false discovery rate adjusted *p* < 0.05, and were selected as PAAD-specific 5hmC-modified lncRNAs. Then a 5hmC-modified lncRNA-based risk scoring model (termed 5hLDS) was constructed using the elastic net regularization on a multivariable logistic regression approaches to distinguish between PAAD and healthy individuals. The 5hLDS was trained with 10-fold cross-validation and optimized using a ROC curve for a grid of parameter values for α and λ (α range, 0.05 to 1.00 with a length = 10; λ range: from 10–1 to 5*10–1 with a 0.1 increment), and this selection process was repeated 20 times. Finally, the 5hLDS was established based on PAAD-specific 5hmC-modified lncRNA markers. The 5hLDS range was 0–1.0 and represented a final probability of PAAD for each sample. The 5hLDS was established using the “trainControl” and “train” functions from the Caret (version 6.0–86) R package.

### Functional Analysis of PAAD-Specific 5hmC-Modified lncRNA Markers

Pearson correlation coefficient was used to measure the expression relevance among PAAD-specific 5hmC-modified lncRNA markers, miRNAs and mRNAs in the TCGA cohort. Human experimentally validated miRNA-mRNA interactions were downloaded from ENCORI (https://starbase.sysu.edu.cn/index.php), the updated version of the StarBase database providing the most comprehensive network of miRNA-mRNA interactions supported by CLIP-Seq data sets (Li et al., 2014). The collected data contain 9,664 experimentally validated miRNA-mRNA interactions, including 276 miRNAs and 14,837 mRNAs. Finally, PAAD-specific 5hmC-modified lncRNA marker-related competitive endogenous RNA (ceRNA) networks were constructed based on ceRNA mechanism as follows: 1) there was a significantly high negative co-expression relationship (*p* < 0.05 and r < -0.2) between miRNA and mRNA, and between miRNA and lncRNAs; 2) there was significantly high positive co-expression relationship (*p* < 0.05 and r > 0.4) between mRNA and lncRNAs; 3) there were experimentally validated miRNA-mRNA interactions; This ceRNA network was visualized using the Cytoscape software (version 3.8.2). Functional enrichment analysis of Gene Ontology (GO) and Kyoto Encyclopedia of Genes and Genomes (KEGG) for mRNAs in the ceRNA network was performed with Metascape (Zhou et al., 2019b).

### Statistical Analysis

A diagnostic model was developed using the machining learning approach, and a prognostic model was developed using the Cox regression model. The diagnostic model was evaluated with cross-validation and ROC methods. The conventional univariate Cox proportional hazards regression model for overall survival data was implemented to identify variables associated with overall survival. Significant factors in univariate analysis were further subjected to a multivariate Cox regression analysis. Hazard ratios (HR) and corresponding 95% confidence interval (CI) were calculated in the Cox models. The optimal risk cut-off value of the prognostic model was calculated using the R package “survminer”. The PAAD patients were stratified into high- and low-risk groups according to the risk cut-off. Kaplan-Meier curve analysis and Log-rank test were performed to visualize and compare the survival difference between the two risk groups. A Multivariate Cox regression model was used to examine whether the prognostic model remained significant after adjusting clinical variables. Statistical significance was performed for categorical variables using the Wilcoxon signed-rank test for two-group comparisons unless otherwise specified in the figure legend. All statistical analysis was conducted in R software, version 3.6.1.

## Results

### Identification of Altered Plasma 5hmC Modifications in lncRNAs Genes Involved in PAAD

**FIGURE 2:**
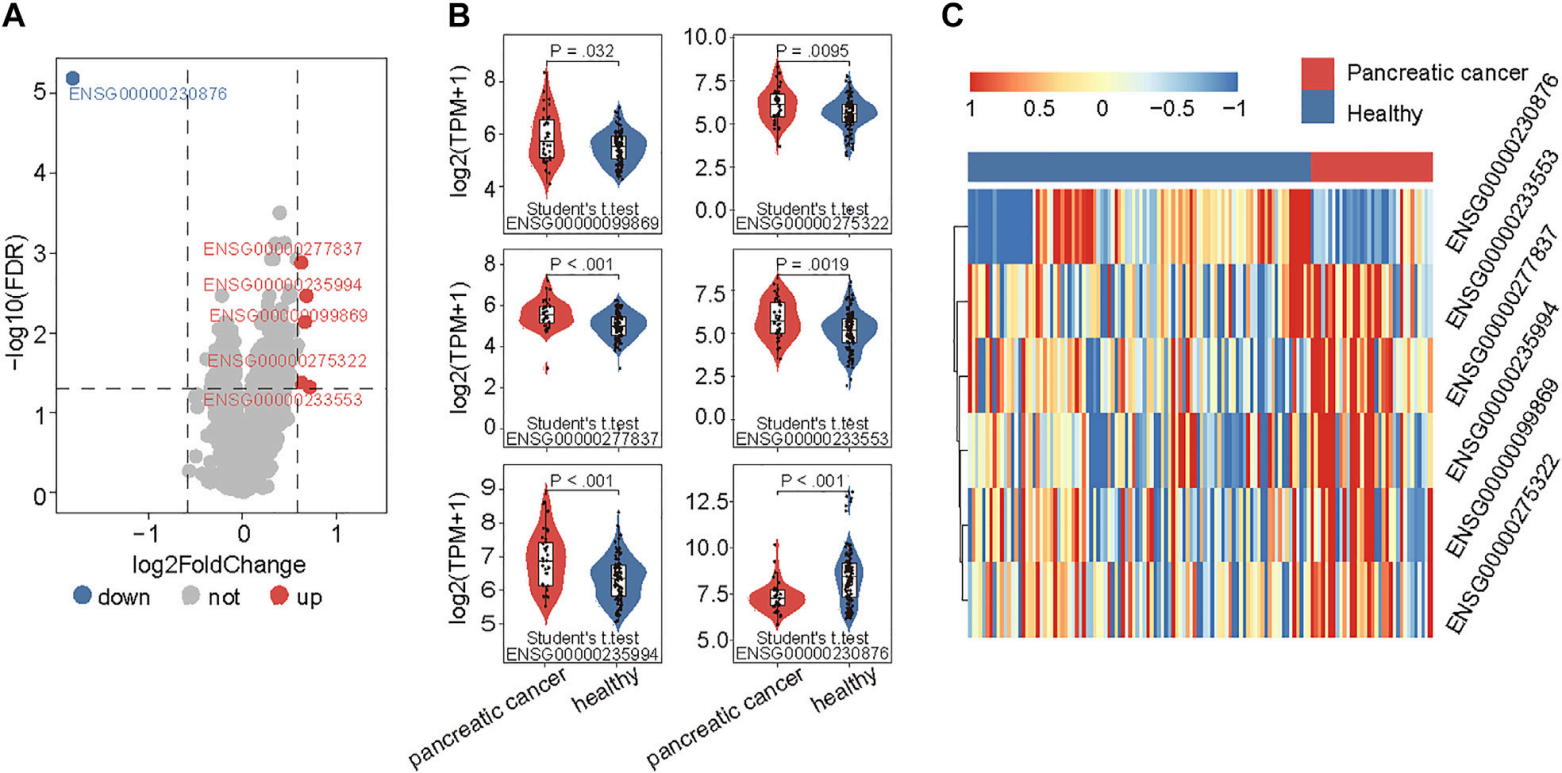
**I**dentification of PAAD-specific 5hmC-modified lncRNAs. **(A)** Volcano plot visualizing the intersections of 5hmC-modified up- and down-enriched lncRNAs in PAAD samples compared with healthy controls. **(B)** Boxplots showing 5hmC modification levels of lncRNAs between PAAD and healthy samples. Statistical significance was determined using the Student’s t-test. **(C)** Heatmap of unsupervised hierarchical clustering of PAAD-specific 5hmC modification levels in lncRNAs. PAAD, Pancreatic adenocarcinoma; lncRNA, long non-coding RNAs; 5hmC, 5-Hydroxymethylcytosine.

To examine the 5hmC modification of lncRNAs genes in PAAD, we compared the 5hmC profiles of lncRNAs between PAAD (*n* = 34) and healthy (*n* = 96) samples and found a total of six lncRNAs that showed differential 5hmC modification patterns (Figure 2A). Among six lncRNAs, five lncRNAs (*RP11-262A16.1*, *IGF2-AS1*, *AC108462.1*, *RP11-714M23.2* and *RP3-470B24.5*) showed increasing 5hmC modification and one lncRNAs (*LINC00486*) showed decreasing 5hmC modification in PAAD compared to healthy samples (Figure 2B). These six lncRNAs with abnormal 5hmC modifications were defined as 5hmC-modified lncRNAs. As shown in Figure 2C, the 5hmC levels of six 5hmC-modified lncRNAs were significantly different and revealed clinical potential as diagnostic biomarkers in distinguishing PAAD from healthy controls (Figure 2C).

### Development and Validation of a Plasma-Derived Diagnostic Model Based on 5hmC-Modified lncRNAs

**FIGURE 3:**
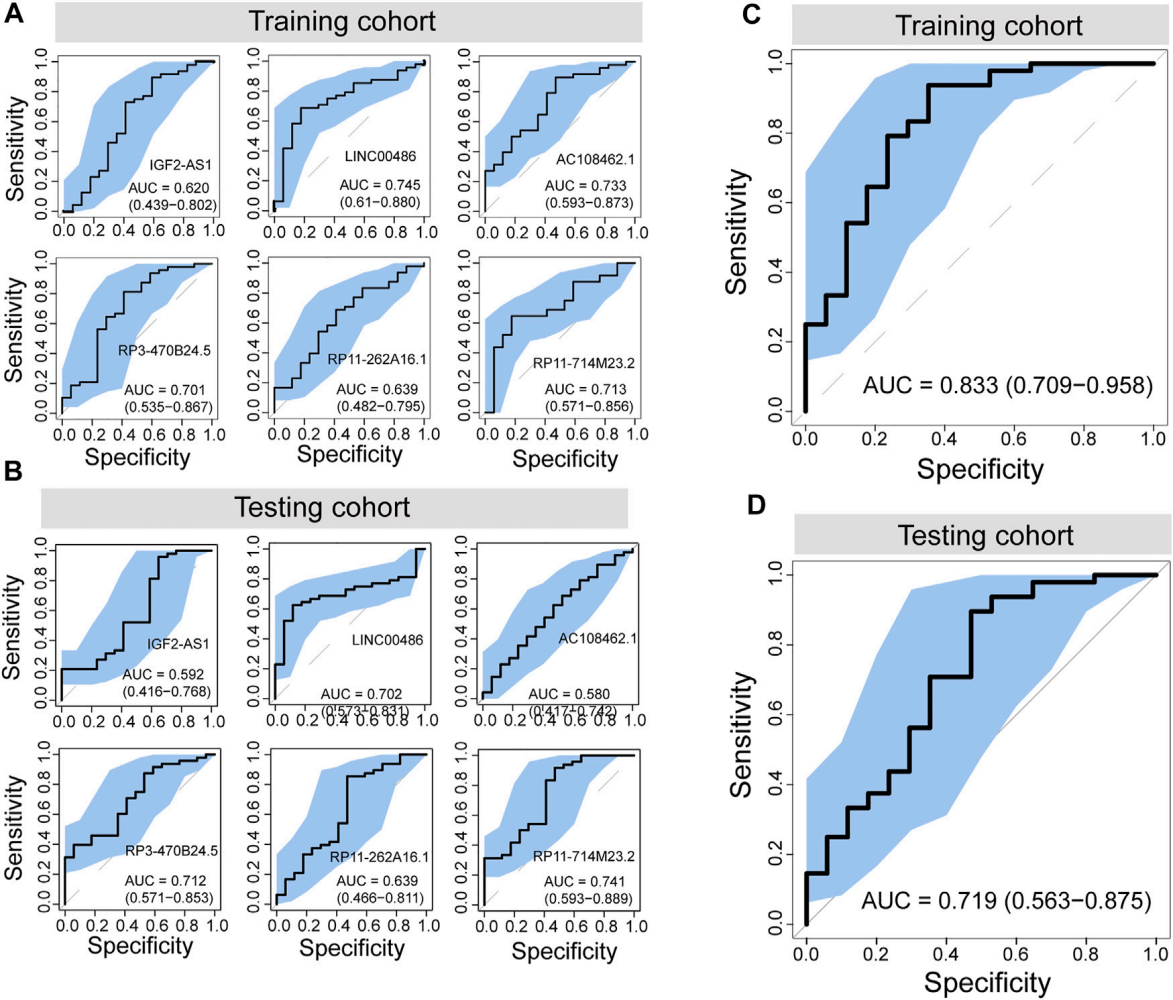
Development and independent validation of a plasma-derived diagnostic model (5hLDS) based on 5hmC-modified lncRNAs. ROC curve and AUC value of each PAAD-specific 5hmC-modified lncRNA for cancer diagnosis in the training cohort **(A)** and testing cohort **(B)**. ROC curve and AUC value of the 5hLDS for cancer diagnosis in the training cohort **(C)** and testing cohort **(D)**. AUC, Area under the ROC curve; PAAD, Pancreatic adenocarcinoma; p5hmC-score, PAAD 5hmC-LncRNA diagnosis score. PAAD, pancreatic adenocarcinoma.

To further evaluate the potential of differentially regulated 5hmC modified lncRNAs as diagnostic biomarkers for PAAD, we conducted a discovery-validation study, in which 130 plasma cfDNA samples were split randomly into equally sized training cohort and testing cohort, respectively. We estimated the contribution of each differentially regulated 5hmC modified lncRNAs to disease diagnosis using the 5hmC-modified levels as the contributed score in the training and testing cohorts. As shown in Figures 3A,B, each of the differentially regulated 5hmC modified lncRNAs exhibited a diagnostic performance AUC of 0.62–0.745 in the training cohort and 0.58 to 0.741, respectively.

Then, we developed a machining learning-based non-invasive diagnostic model based on six 5hmC modified lncRNAs using the elastic net regularization on a multivariable logistic regression approach (termed 5hLDS) in the training cohort. Using the ten-cross-validation, the 5hLDS could discriminate PAAD from healthy individuals with an overall AUC of 0.833 (95% CI 0.709-0.958) (Figure 3C). Then the 5hLDS was validated in the independent testing cohort and yielded a classification performance AUC of 0.719 (95% CI 0.563-0.875) for PAAD detection (Figure 3D). At a disease risk cut-off of 0.5, the 5hLDS achieved 81.54% (95% CI 69.97%-90.08%) and 78.46% (95% CI 66.51%-87.69%) of accuracy to distinguish PAAD patients from healthy individuals in the training cohort and testing cohort, respectively. These results demonstrated AUC improvement when incorporated six differentially regulated 5hmC modified lncRNAs into the diagnostic model.

### Association Between 5hmC-Modified lncRNAs and Prognosis

**FIGURE 4:**
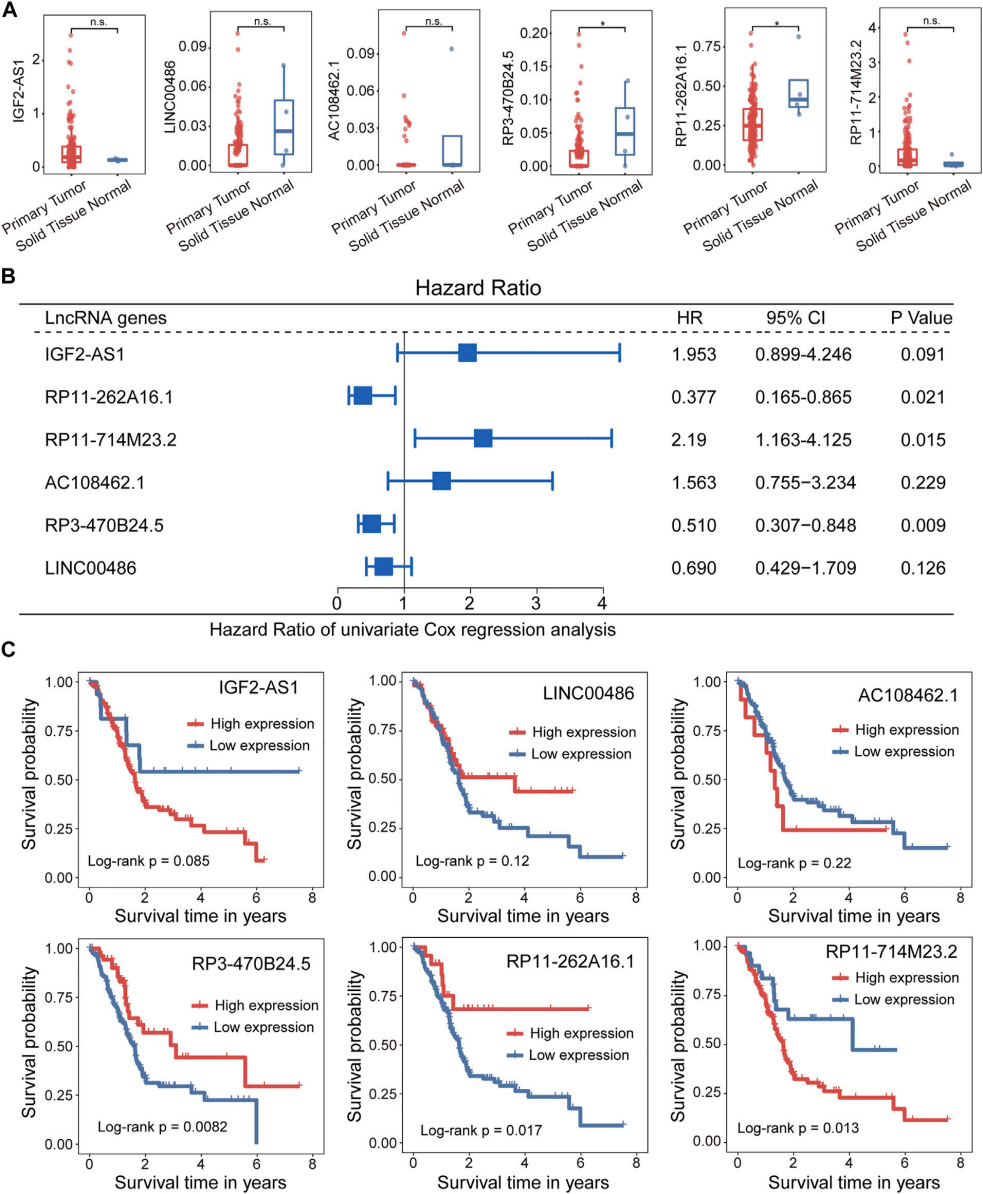
Association between the 5hLD-score and disease outcome. **(A)** Boxplots showing the distribution of expression levels of lncRNAs between PAAD patients and healthy controls. Statistical significance was determined using Wilcoxon signed-rank test. **(B)** Metaplot of univariate analysis of 5hmC-modified lncRNA between high-expression and low-expression group. **(C)**. Kaplan-Meier survival curves of each PAAD-specific lncRNA between high-expression and low-expression groups. PAAD, pancreatic adenocarcinoma.

We further examined whether expression levels of 5hmC-modified lncRNAs were dysregulated in PAAD compared to controls. Expression profiles of 5hmC-modified lncRNAs were obtained from TCGA and were compared between PAAD primary tumors and normal solid tissues. Two of six 5hmC-modified lncRNAs exhibited differential expression patterns between PAAD primary tumors and normal solid tissues. As shown in Figure 4A, lncRNAs RP3-470B24.5 and RP11-262A16.1 revealed significant or marginally significant downregulated expression levels in PAAD compared to normal solid tissues. Furthermore, three of six 5hmC-modified lncRNAs showed significant association correlation between expression levels and OS, including *RP11-262A16.1* (HR = 0.377, 95% CI 0.165–0.865, *p* = 0.021), *RP11-714M23.2* (HR = 2.190, 95% CI 1.163–4.125, *p* = 0.015) and *RP3-470B24.5* (HR = 0.51, 95% CI 0.307–0.848, *p* = 0.009) (Figure 4B). Kaplan-Meier survival analysis showed that PAAD patients with high expression of *RP3-470B24.5* and *RP11-262A16.1* have significant improved OS compared to those with low expression (log-rank test *p* < 0.0082 for *RP3-470B24.5* and *p* < 0.017 for *RP11-262A16.1*), while PAAD patients with high expression of *RP11-714M23.2* tended to have poor OS compared to those with low expression (log-rank test *p* < 0.013) (Figure 4C). These results demonstrated the potential of three 5hmC-modified lncRNAs as prognostic biomarkers.

### 5hmC-Modified lncRNAs-Based Prognostic Prediction Model for PAAD

**FIGURE 5:**
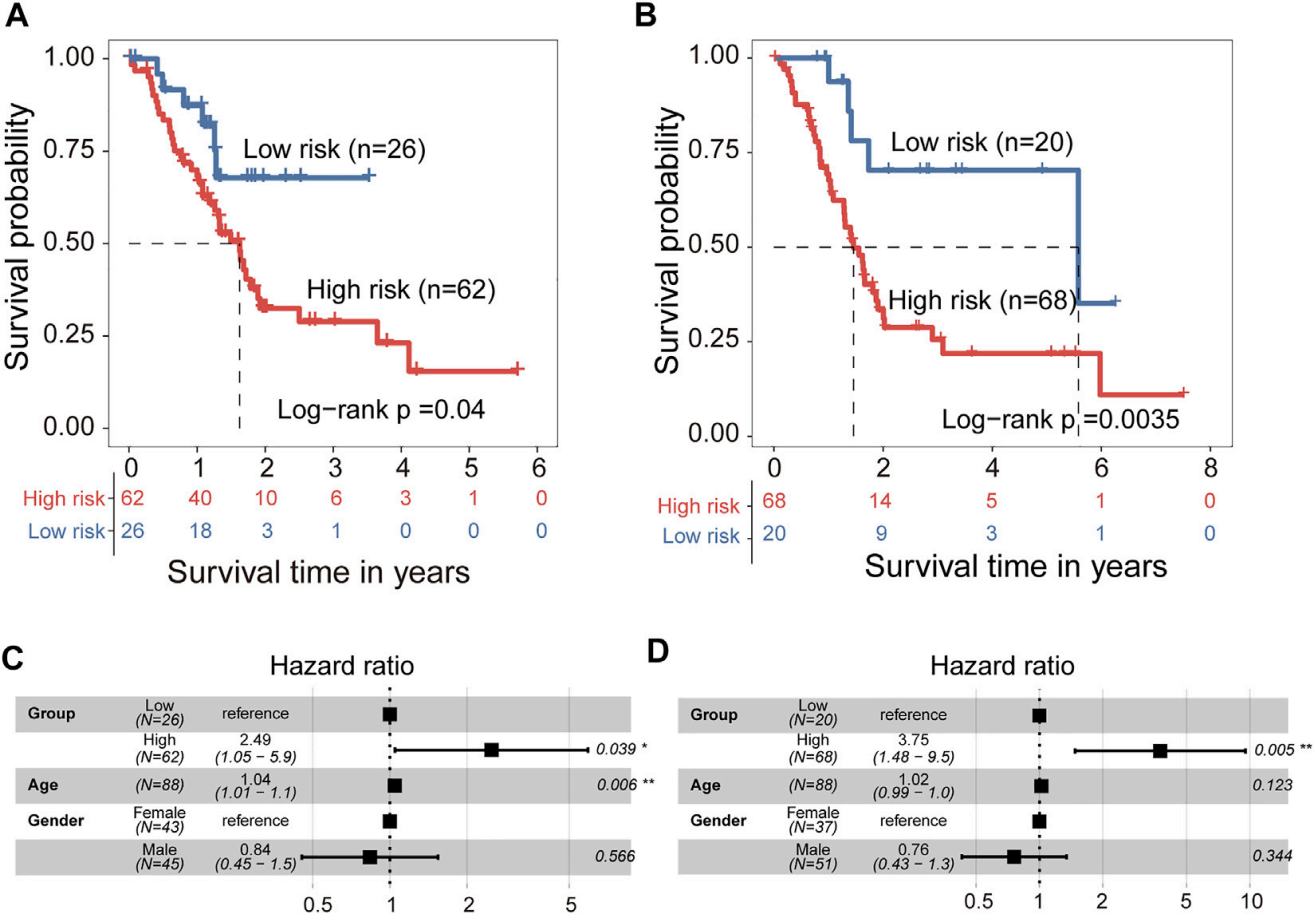
Development and independent validation of 5hmC-modified lncRNAs-based prognostic prediction model (5hLPS). Kaplan-Meier survival curves of patients between the high-risk and low-risk groups in training cohort **(A)** and testing cohort **(B)**. Forest plot of HR deriving from multivariate Cox regression analysis of 5hLPS with other clinical characteristics in training cohort **(C)** and testing cohort **(D)**. HR, Hazard ratios.

We developed a 5hmC-modified lncRNAs-based prognostic risk score model (5hLPS) for the prognostication of PAAD using the linear combination of the expression of three 5hmC-modified lncRNAs and weighted by relative coefficients in the multivariate Cox regression as follows: 5hLPS = (-0.24378700) × expression value of *RP11-262A16.1* + 0.02015193 × expression value of *RP11-714M23.2* + (-1.97034856) × expression value of *RP3-470B24.5*. Then we also conducted a discovery-validation study by splitting PAAD samples from TCGA into equally sized training (n = 88) and testing (*n* = 88) cohort. The optimal risk cut-off value of the 5hLPS stratified 88 patients of the training cohort into the high-risk group (*n* = 62) and low-risk group (*n* = 26) with significantly different OS. As shown in Figure 5A, patients in the high-risk group had significantly shorter OS time than those in the low-risk group (log-rank test *p* = 0.04). When validated in the testing cohort, the 5hLPS separated the 88 patients of the testing cohort into high-risk (*n* = 68) and low-risk groups (*n* = 20). The median survival time in the low-risk group was significantly better than that in the high-risk group (log-rank test *p* = 0.0035) (Figure 5B). The univariate Cox analysis also showed that the 5hLPS has a significant association with OS both in the training (HR = 2.4, 95% CI: 1.0–5.7, *p* < 0.05) and testing (HR = 3.6, 95% CI: 1.4–9.2, *p* < 0.01) cohorts. To further examine whether the 5hLPS was independent of other clinical and pathological factors. We performed multivariable Cox proportional hazards analysis, including individual clinical variables with the 5hLPS in each cohort. As shown in Figure 5C, in the training cohort, the 5hLPS (HR = 2.49, 95% CI 1.05–5.9, *p* < 0.039) and age (HR = 1.04, 95% CI 1.01–1.1, *p* = 0.006) were significantly associated with OS in the multivariate analysis. In the testing cohort, only the 5hLPS maintained a significant association with OS (HR = 3.75, 95% CI: 1.48–9.5, *p* = 0.005) (Figure 5D). These results suggested that 5hLPS is an independent predictive factor for patients with PAAD.

### Functional Characterization of 5hmC-Modified lncRNA Markers

**FIGURE 6:**
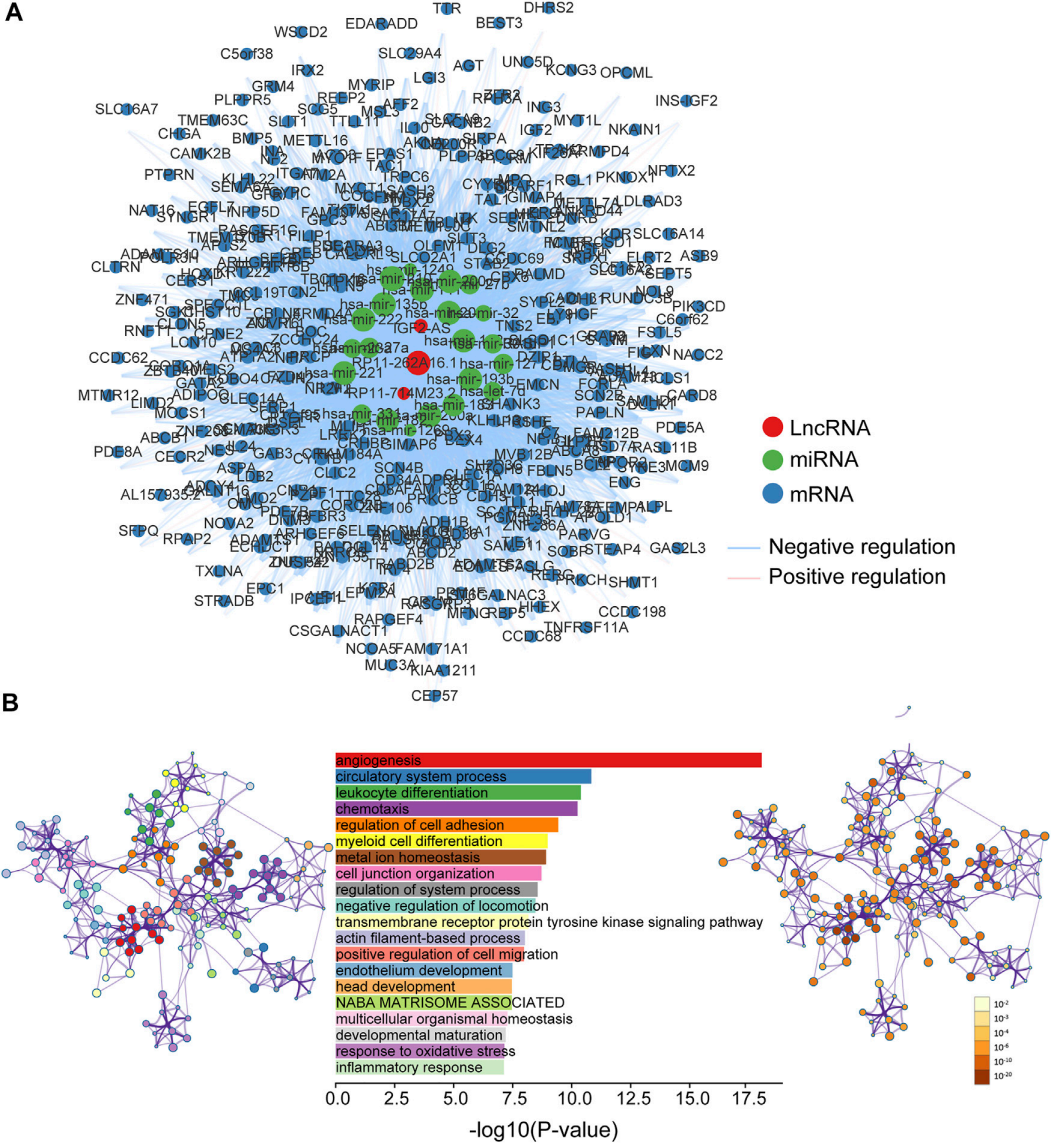
Functional characterization of PAAD-specific 5hmC-modified lncRNAs. **(A)** PAAD-specific 5hmC-modified lncRNA-associated ceRNA network. Blue balls represent mRNAs; red balls represent lncRNA; green balls represent miRNA. Blue edges indicate lncRNA-miRNA-mRNA negative interactions; pink edges indicate lncRNA-miRNA-mRNA positive interactions. The size of the shapes represents the degrees that are involved. The bigger the shape, the higher the degree. **(B)** Bar graph of enriched terms across mRNA gene lists, length by -log10(P-value). Network graph of the enriched term (left pannel) colored by cluster-ID, where nodes that share the same cluster-ID are typically close to each other; and enriched *p*-value (right panel) colored by *p*-value, where terms containing more genes tend to have a more significant *p*-value. miRNA, microRNA; lncRNA, long non-coding RNA; ceRNA, competitive endogenous RNA; PAAD, pancreatic adenocarcinoma.

To explore the functional roles of 5hmC-modified lncRNAs, we constructed a 5hmC-modified lncRNA marker-associated ceRNA network which included 5,604 interactions among 358 mRNAs, three lncRNAs and 22 miRNAs (Supplementary File S1), as shown in Figure 6A. Then we performed pathway functional enrichment analysis for 358 mRNAs in this ceRNA *via* Metascape to infer potential functional roles of 5hmC-modified lncRNA markers. As shown in Figure 6B, 5hmC-modified lncRNA marker-associated mRNAs were significantly enriched in angiogenesis, circulatory system process, leukocyte differentiation, and metal ion homeostasis, which are known important events in the development and progression of the development and progression of PAAD.

## Discussion

Pancreatic adenocarcinoma (PAAD) has an inferior prognosis and remains a lethal malignancy (Schizas et al., 2020). Recent studies suggest that aberrant expression of lncRNA drives the initiation and progression of malignancies (Bhan et al., 2017). Liquid biopsies provide a non-invasive approach to detect tumors (Shen et al., 2018) and a novel Nano-hmC-Seal technology to generate the genome-wide profiles of 5hmC in cfDNA from blood plasma for multiple cancer types (Li et al., 2017). Whether plasma-derived 5hmC-modified lncRNA is a conclusive biomarker for distinguishing the type of cancer and diagnosing cancer is unclear. In this study, we explored the potential application of the plasma-derived 5hmC modification level in lncRNA being used as an alternative biomarker for PAAD diagnosis and monitoring.

In this study, by redefining 5hmC sequencing reads to lncRNA genes, 5hmC alterations of lncRNAs were characterized in PAAD. Several altered 5hmC modifications were distributed at lncRNAs in patients with PAAD compared with healthy subjects. Herein, these differentially regulated 5hmC modified lncRNAs were considered as PAAD-specific markers. We trained machine-learning algorithms with 10-fold cross-validation using the differentially regulated 5hmC modified lncRNAs as features (termed 5hLDS), and evaluated the prediction performance in the training and testing cohorts. The 5hLDS achieved superior diagnostic performance in distinguishing PAAD from healthy controls both in the training and testing cohorts.

Further exploring of expression relevance of the 5hmC-modified lncRNAs in PAAD identified two of six 5hmC-modified lncRNAs that were dysregulated expression in PAAD compared to normal tissues, suggesting that these 5hmC modifications changes might lead to dysregulated lncRNAs expression that involved in the development of PAAD. Furthermore, we further investigated the effect of expression of 5hmC-modified lncRNAs on survival and found that 5hmC-modified lncRNAs were also able to make a distinction between each PAAD patient into distinct groups with better or worse survival outcomes. These results also support the idea that 5hmC-modified lncRNAs can serve as potential biomarkers for the prognosis of PAAD patients. Therefore, we trained a regression model with Cox analysis using the differentially regulated 5hmC modified lncRNAs as features (termed 5hLPS), and evaluated the prognostic performance in the training and testing cohorts. The 5hLPS exhibited superior performance in classifying the patients into two groups with significantly different overall survival independent of clinical variables.

Although many lncRNAs have been discovered and recorded in a vast amount of literature, only a few have been well-functionally studied and characterized. It has been reported that lncRNAs can act as ceRNA for miRNA and emerge as important regulators involved in diverse biological and physiopathological contexts. Among six 5hmC-modified lncRNAs identified in this study, lncRNA *IGF2-AS1* is expressed in antisense to the insulin-like growth factor 2 (IGF2) gene and is imprinted and paternally expressed (Vu et al., 2003). Many studies suggested that transcripts from *IGF2-AS1* are produced in tumors and may suppress cell growth (Unger et al., 2017; Kasprzak and Adamek, 2019; Contractor et al., 2020). Another 5hmC-modified lncRNAs, *LINC00486*, has been reported to be a hot spot of breakpoints and has a high rearrangement rate in non-small cell lung cancer cells (Wang et al., 2018). A recent study also found that *LINC00486* may act as a tumor suppressor gene and its overexpression can inhibit the proliferation and promote the apoptosis of breast cancer tissues by suppressing miR-182–5p expression (Yuan et al., 2020). To further gain a novel functional insight for 5hmC-modified lncRNAs, we construed a 5hmC-modified lncRNAs-associated ceRNA network according to the ceRNA hypothesis and found that mRNA in this ceRNA network were involved in the known crucial event in the development and progression of PAAD, such as angiogenesis, circulatory system process, leukocyte differentiation and metal ion homeostasis.

The present study still had its limitations. First, this study did not obtain significant clinical variables of 5hmC-modified lncRNAs, such as follow-up time. Further independent validation studies should be performed to help address problems such as the potential selection bias for model construction. Secondly, the regulatory mechanism of 5hmC and lncRNA genes remains unclear due to the lack of paired 5hmC profiles and lncRNAs expression profiles. Finally, although the 5hmC-modified lncRNAs have been validated in expression levels from the TCGA cohort, further validation is required in other retrospective or prospective cohorts to demonstrate the generalizability of 5hmC-modified lncRNA for PAAD diagnosis and prognosis.

## Conclusion

This study characterized the genome-wide pattern of lncRNA-associated plasma cfDNA 5hmC changes in PAAD, and demonstrated potential roles for 5hmC in the transcriptional regulation of lncRNAs contributing to the development and progression of PAAD. Finally, we identified and validated 5hmC-modified lncRNA panels for the diagnosis and prognosis of PAAD with high and robust performance, which presented potential clinical utility of 5hmC-modified lncRNAs as valuable biomarkers for non-invasive diagnosis and prognostication of cancers.

## Data Availability

Publicly available datasets were analyzed in this study. This data can be found here: The datasets of this article are available in the Sequence Read Archive repository (https://trace.ncbi.nlm.nih.gov/Traces/sra/?study=SRP080977) and UCSC Xena (https://xena.ucsc.edu/).
